# Ultrasound guided chest compressions during cardiopulmonary resuscitation

**DOI:** 10.1186/2036-7902-7-S1-A32

**Published:** 2015-03-09

**Authors:** P Benato, M Zanatta, V Cianci

**Affiliations:** Emergency Department of Arzignano Hospital, ULSS5 Ovest Vicentino, Vicenza, Italy

**Keywords:** Left Ventricle, Cardiac Arrest, Cardiopulmonary Resuscitation, Chest Compression, Hand Position

## Background

Early and effective chest compressions have a well known pivot role in cardiopulmunary resuscitation (CPR) and 2010 International Consensus on Cardiopulmonary Resuscitation have strongly reinforced its importance.

The efficacy of chest compressions depends on hands position and on compression technique.

Medical education can improve chest compression technique, while the choice of thoracic landmark is always blind even if 2010 consensus indicated that it is reasonable to place the hands in the lower half of the sternum.

## Objective

Critical care ultrasound (CCUS) has changed the approach of critical ill patient and can identify potential reversible causes of cardiac arrest during CPR.

Our challenge is to use CCUS to locate the most appropriate site for chest compressions.

## Matherial and method

We planned a pilot study (in progress) to evaluate the capability of CCUS to improve the quality of chest compressions while CPR is taking place.

## Results

We describe data of a small case series from 6 non traumatic cardiac arrests who was treated both in-hospital and in pre-hospital settings.

In 3 out of 6 patients compressions were correctly performed while in the other 3 cases partials left ventricle compression or the narrowing of the base of the heart and aorta was observed. Ultrasound guided changes in hands position improved passive left ventricle contractility in the 3 incorrect CPR.

## Conclusions

Our study doesn’t permit to estimate if the changes made in hands position would have affected the outcome of CPR.

Anyway we think that the possibility to focus the power of the hands over the real position of left ventricle certainly improves the quality of our chest compressions.
